# Platelet-Rich Fibrin in Non-Surgical Periodontal Therapy: A Split-Mouth Randomized Controlled Clinical Trial

**DOI:** 10.3390/dj12050135

**Published:** 2024-05-09

**Authors:** Simran R. Parwani, Kaustubh S. Thakare, Kshipra P. Kawadkar, Nishita Jaju Soni, Rajkumar Parwani, Himanshu Dadlani, Dhanashree S. Chaudhary, Dipanshu Pahuja, Gianrico Spagnuolo, Niccolò Giuseppe Armogida

**Affiliations:** 1 Department of Periodontology, V.Y.W.S. Dental College and Hospital, Amravati 444602, India; simpar74@gmail.com (S.R.P.); kaustubh.thakare.mds@gmail.com (K.S.T.); kshipra.kawadkar@gmail.com (K.P.K.); dr.nishita.manmohan.soni@gmail.com (N.J.S.); dhana.shree.chaudhari.2710@gmail.com (D.S.C.); dipanshu.pahuja.1@gmail.com (D.P.); 2 Department of Oral Pathology, V.Y.W.S. Dental College and Hospital, Amravati 444602, India; dr_rnparu@yahoo.co.in; 3 Department of Periodontology, Kalka Dental College, Meerut 250103, India; himdent@hotmail.com; 4 Principal Consultant, Department of Dentistry, Max Hospital, Gurugram 122001, India; 5 Department of Neurosciences, Reproductive and Odontostomatological Sciences, University of Naples Federico II, 80131 Naples, Italy; ng.armogida@gmail.com; 6 Therapeutic Dentistry Department, Institute for Dentistry, Sechenov University, Moscow 119991, Russia

**Keywords:** platelet-rich fibrin, periodontal pocket, periodontitis, scaling and root planing

## Abstract

This clinical trial investigated the efficacy of platelet-rich fibrin (PRF) as an adjunct to conventional scaling and root planing (SRP) in non-surgical periodontal therapy. In a split-mouth randomized controlled trial with 13 patients and 26 periodontal pocket sites, PRF was inserted in test group pockets alongside SRP, while control group pockets received SRP alone. Measurements at baseline and six weeks included probing pocket depths (PPDs), clinical attachment loss (CAL), gingival recession (GR), the plaque index, and the gingivitis index. The wound healing index was assessed at six weeks. The results show statistically significant improvements in the SRP+PRF group compared to SRP alone, demonstrating a better CAL gain (SRP+PRF group: 2.69 ± 0.63; SRP alone group: 4.15 ± 0.69—*p*-value: 0.001), PPD reduction (SRP+PRF group: 2.62 ± 0.65; SRP alone group: 3.85 ± 0.80—*p*-value: 0.001), and GR minimization (SRP+PRF group: 0.46 ± 0.62; SRP alone group: 0.81 ± 0.72—*p*-value: 0.21). The adjunctive use of PRF enhanced healing, reduced pocket depths, decreased tissue morbidity, and minimized gingival recession. This study concludes that PRF placement is effective in 5–6 mm pockets, potentially reducing the number of periodontal treatment sessions needed for pocket closure.

## 1. Introduction

Periodontitis is defined as a multifactorial infectious disease that occurs due to the challenge between the host response and specific periodontal pathogens and is characterized by a slow destruction of periodontal supporting tissue in during a period of time [1,2,3]. Indicators of the loss of periodontal tissue are evidenced by the clinical attachment loss (CAL) and radiographically assessed alveolar bone loss, along with the presence of periodontal pocketing and gingival bleeding [4,5].

The pathophysiology of periodontitis involves a chronic, multifactorial inflammatory disease associated with dysbiotic dental plaque biofilms [5]. It stands as the most common chronic inflammatory noncommunicable disease in humans. In particular, the prevalence of an advanced and severe form of periodontitis was estimated to be 7.4% [6]. Additionally, milder forms of periodontitis may have a prevalence as high as 50% [7].

Left untreated, it can lead to tooth loss, although in the majority of cases, it is preventable and treatable [5].

The aim of the periodontal treatment is the supra- and subgingival removal of the pathogenic biofilm (Professional Mechanical Plaque Removal—PMPR) and the improvement of the oral health and hygiene behavior. Hence, the initial phase of the PMPR involves manual and/or ultrasonic supragingival hygiene. This is succeeded by Step 2 of the therapy, referred to as cause-related therapy, which endeavors to diminish or eradicate subgingival biofilm and calculus through scaling and root planing (SRP). The SRP procedure can be executed using hand or powered instruments, including sonic or ultrasonic devices, either independently or in tandem [5]. Notably, recent advancements have introduced alternative techniques such as laser-based approaches [8,9]. This is an essential step performed in all periodontal therapies that determinates an average of a 1.7 mm of pocket reduction [5]. Therefore, periodontal pockets deeper than 5 mm, accompanied by radiographic evidence of infrabony defects, warrant treatment through a surgical approach [5].

After PMPR and other procedures required to remove all etiologic factors responsible for the accumulation of plaque, at least six/eight weeks are needed for the expected healing of periodontal tissues [5,10]. After about 2 months from the non-surgical approach, residual pocket depths can be measured to evaluate the need for surgical procedures [5]. This time can be utilized for the proper education and motivation of patients to improve their home care skills for reducing gingival inflammation and inculcate new habits that will ensure the success of the treatment [5]. Additional betterment from these therapies can be expected only if phase I therapy results in gingival tissues are free of frank inflammation and the patient has developed efficient regular plaque control measures [10].

Therefore, the complete regeneration of hard and soft tissues is the primary aim of periodontal regenerative treatment. In this context, a variety of techniques and materials have been suggested to obtain the complete healing/regeneration of lost periodontal support [10]. Guided Tissue Regeneration (GTR) is a surgical procedure used to achieve this goal. It is performed using bone grafting, enamel matrix proteins, and membrane, used alone or in combination with each other [9]. Alternative surgical techniques incorporate stem cell application, the modulation of the host response, and platelet concentration to enhance the success of the regeneration [11,12,13,14,15,16,17]. However, it is important to note that wound healing following periodontal surgery can be adversely impacted by factors such as surgical incision, periosteal disruption, and flap elevation [16,17].

Surgical treatment is expensive and needs to be performed by an expert clinician with great manual skills. In order to exceed the limitations of periodontal surgery, various alternative non-surgical treatment modalities like cell- and gene-based techniques, applications of stem cells, a modulated host response, and the employment of platelet concentrates can be used [18].

Platelet-rich plasma (PRP) is a platelet concentrate developed in 1988 and used as an adjunctive component to enhance periodontal healing [19]. PRP has emerged as a promising biological agent in combination with bone graft materials for periodontal-guided tissue regeneration therapy. It has demonstrated significant efficacy in achieving clinical outcomes such as CAL gain, reduction in probing depth (PD), and the recovery of lost bone [20,21]. Nevertheless, it is noteworthy that when compared to other biologic agents like platelet-rich fibrin, PRP tends to yield less optimal results in terms of CAL gain, PD reduction, bone gain, and recession depth reduction [21].

PRF is a second-generation platelet concentrate free of anticoagulant and thrombin [12,22] and could be shaped as membrane, helping in the regeneration of lost soft and hard tissues and contributing as a potential biologic response modifier. PRF serves as a scaffold, preventing the premature migration of epithelial cells into the periodontal tissues [23,24]. The regeneration capacity of PRF is due to its angiogenetic potential, which can be explained by the 3D fibrin matrix that can carry, at the same time, cytokines and growth factors such as Vascular Endothelial Growth Factor (VEGF), Insulin Growth Factor (IGF), Transforming Growth Factor β1 (TGF-β1), and Platelet-Derived Growth Factor (PDGF) [25,26].

The present study aimed to evaluate the clinical parameters in periodontitis patients after Step 2 of the periodontal treatment with a concomitant application of PRF membrane in moderate-depth periodontal pockets compared to the conventional Step 2 of therapy [5].

## 2. Materials and Methods

### 2.1. Study Sample

This study was a split-mouth randomized controlled clinical trial. Systemically, healthy patients with an age range from 30 to 60 years old with probing pocket depth (PPD) ranges from 5 to 6 mm and willing to participate in the research were selected from the Outpatient Department, Periodontology, VYWS Dental College and Hospital, Amravati. Patients were classified as Stage III Grade A periodontitis according to the 2017 periodontal disease classification [27]. The present study was structured as a split-mouth trial to thoroughly examine the varying responses of individual hosts to two different treatment approaches. This study was registered on Clinical Trial PRS, Protocol Registration and Results System, no. NCT05908929, on 16 June 2023, as a retrospective trial registration.

The primary outcome of this study was to define if SRP therapy with the application of PRF membrane could enhance clinical results in terms of PPD reduction, CAL gain, the plaque index [28], the Gingival Index [29] and GR minimization compared with SRP therapy alone.

The secondary outcome was to evaluate the healing ability of the PRF membrane using the wound healing index [30].

### 2.2. Eligibility Criteria

Patients enrolled in the present split-mouth randomized controlled clinical trial were identified through clinical charts presenting periodontitis classified as Stage III Grade A and with 5 or 6 mm periodontal pocket seeking treatment.

The inclusion criteria were as follows: (a) either gender, (b) aged 18 years or older, (c) without a medical history of systemic diseases, (d) with periodontitis Stage III Grade A diagnosed according to the Classification of Periodontal and Peri-Implant Diseases and Conditions 2018 [4], and (e) with a 5 or 6 mm probing depth.

The exclusion criteria were as follows: (a) with medical history of systemic disease or bleeding disorders, (b) the presence of other gingival diseases (such as leukoplakia, lichen planus, pemphigoid disorders, pemphigus vulgaris, herpetic lesions, Necrotizing Ulcerative Periodontitis (NUP)), (c) pregnancy, (d) history of any drug usage affecting the periodontium for the past six months (such as systemic antibiotic therapy), (e) prior periodontal treatment within the preceding six months, (f) smoking, (g) teeth with untreated caries, (h) endodontic lesions and grade II or more mobility, (i) with acute exacerbation of periodontitis, (j) with a systemic disease or condition that could affect tissue healing (e.g., autoimmune disease), (k) severe furcation involvement (grade II and III), (l) abutment for prosthetic rehabilitation, and (m) active orthodontic therapy.

The study protocol was approved by the Ethical Board of the Dental Institutional Research Committee with reference number DCA/IEC/102/2022. Written informed consent forms were duly signed by all patients, and randomization was performed using the coin toss method.

### 2.3. Clinical Indices

Patients enrolled underwent a comprehensive intra- and extra-oral examination. A comprehensive periodontal examination was performed encompassing site-specific pre- and post-operative clinical parameters, such as the plaque index (PI) described by Turesky et al. [28], gingivitis index (GI) described by Loe and Silness [30], probing pocket depth (from the gingival margin to the base of the pocket), clinical attachment loss (CAL—from the cemento-enamel junction to the base of the pocket), and gingival recession (GR—from the gingival margin to the cemento-enamel junction), were measured at baseline and after six weeks post-operatively.

The plaque index was used to assess the presence and the amount of plaque on the teeth that would be treated with the following scoring criteria [28]:

0 = Absence of microbial plaque;
1= Thin film of microbial plaque along free gingival margin;
2 = Moderate accumulation with plaque in sulcus;
3 = Large amount of plaque in sulcus or pocket along the free gingival margin.

To define the grade of gingival inflammation, the gingivitis index was used with the following scoring criteria [29]:

0 = Absence of inflammation;
1= Mild inflammation—slight changes in color and texture;
2 = Moderate inflammation—moderate glazing, redness, edema, or hypertrophy;
3 = Severe inflammation—marked redness and hypertrophy.

The probing pocket depth, clinical attachment loss, and gingival recession were measured with the North Carolina Probe (Hu-Friedy, LLC, Chicago).

The wound healing was classified according to the wound healing index described by Landry, Turnbull, and Howley [30]. This index is based on the tissue color, response to palpation, granulation tissue, and incision margin at six weeks post-operation only and is scored with the following scoring criteria:

Grade 1 (very poor);
Grade 2 (poor);
Grade 3 (good);
Grade 4 (very good);
Grade 5 (excellent).

### 2.4. Sample Size

The outcome used for the power analysis was he probing depth (PD).

The sample size was determined using G Power (v3.1.9.2) software from the data (PD values) obtained from a previous study conducted by Ozcan et al. [17]. The power of this study was calculated prior to the initiation of the study. To obtain 85% power and to compare differences between the means of the probing depth of the two groups using the student’s ‘*t*’ test, 13 sites per group were needed. In total, 26 sites were included.

### 2.5. Clinical Procedure

**Figure 1:**
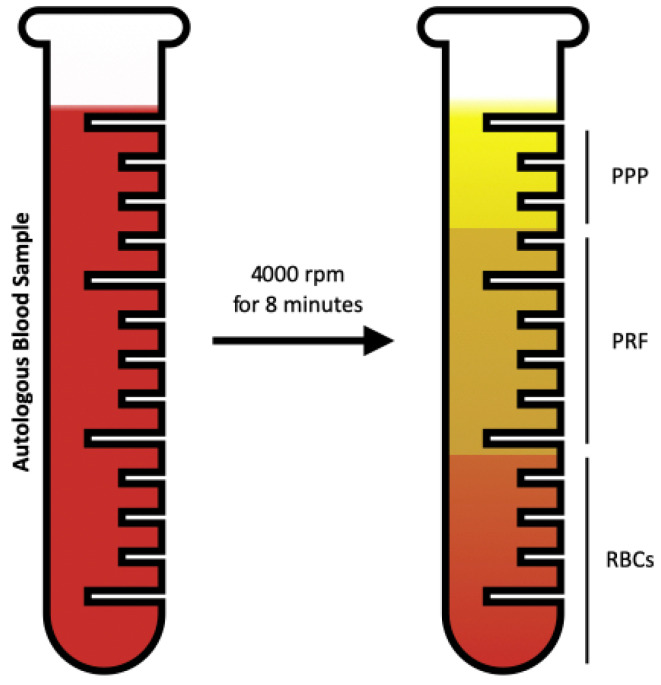
PRF preparation.

Systemically, healthy patients with Stage III grade A periodontitis, with a total of 26 periodontal pocket sites, were selected, having a 5 to 6 mm probing depth and two quadrants with contralateral sides, and randomly assigned to a test and control group using the coin toss method. After completing clinical and radiographic evaluations, patients were treated for their periodontal disease. The periodontal charting was performed using the North Carolina Probe (Hu-Friedy, LLC, Chicago, IL, USA). The periodontal treatment was performed by the same operator for all the patients. Both the test and control group underwent the SRP with a Full-Mouth Disinfection Protocol. Gracey curette and After five Gracey curette (Hu-Friedy, LLC, Chicago, IL) and ultrasonic debridement (Piezo Master 400, EMS^®^, Nyon, Switzerland) were used to perform the SRP. The experimental group involved pockets treated with SRP with the concomitant insertion of PRF membrane. Blood (40 mL) was collected from each patient in four dry glass tubes of 10 mL each. The blood collection was performed swiftly, and the tubes were promptly centrifuged at 4000 rpm for 8 min using a dedicated centrifuge (PC-02, Process for PRF, Nice, France) at room temperature. After the centrifugation, the tube exhibited three distinct parts: the upper one containing Platelet-Pure Plasma (PPP), the middle one composed of a PRF fibrin clot, and the lower containing red blood cells (RBCs) (Figure 1).

**Figure 2:**
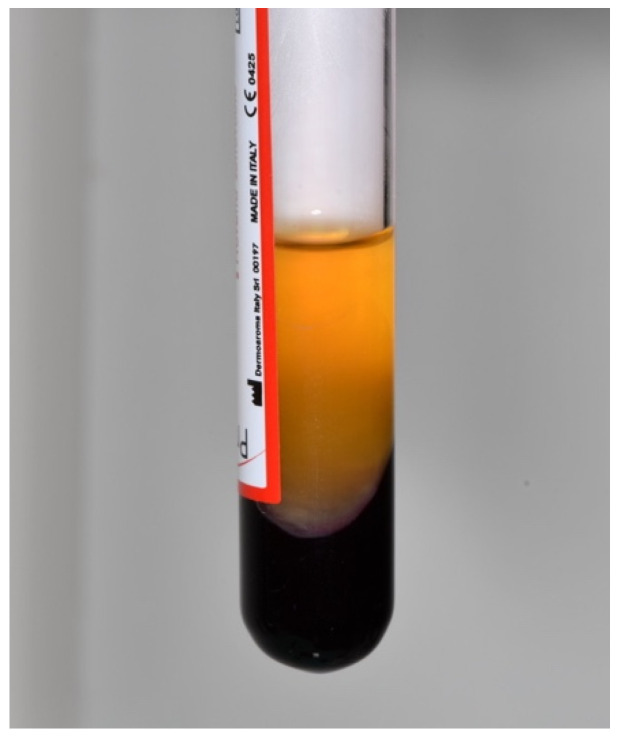
Sterile plastic tubes with the centrifuged blood sample.

**Figure 3:**
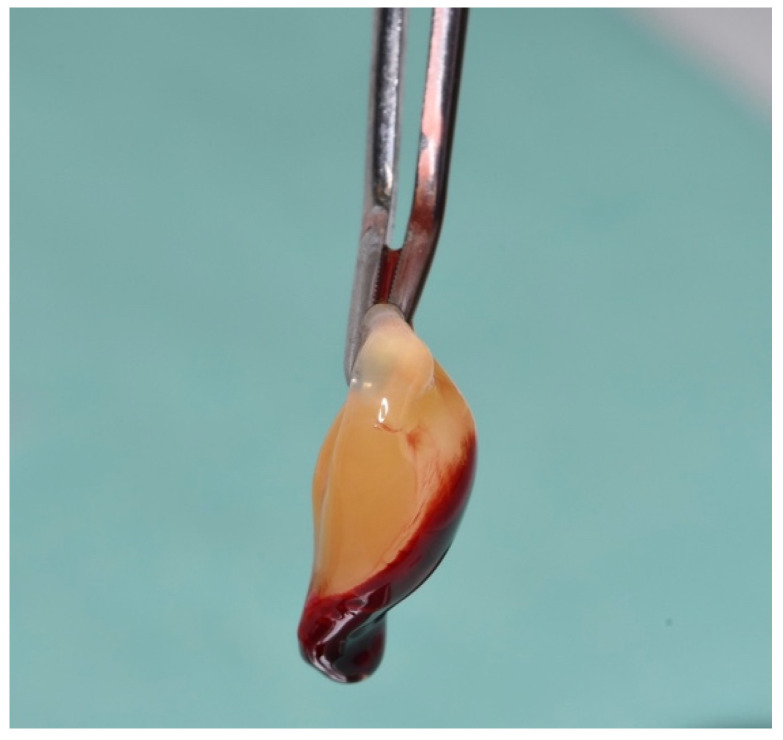
PRF fibrin clot.

**Figure 4:**
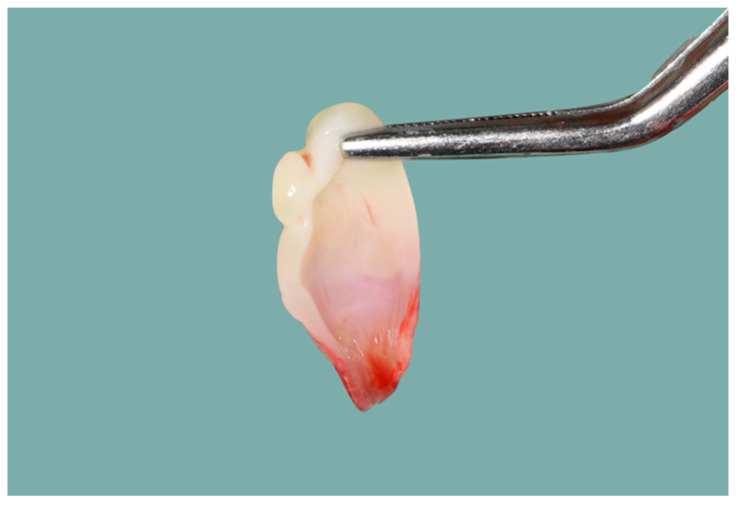
PRF fibrin membrane.

The PRF fibrin clot was delicately extracted from the plastic tube using sterile tweezers (as shown in Figure 2 and Figure 3), isolated from the red blood cells using scissors, and subsequently placed into a sterile cup. The PRF was then converted into a membrane using a specialized container (Figure 4).

**Figure 5:**
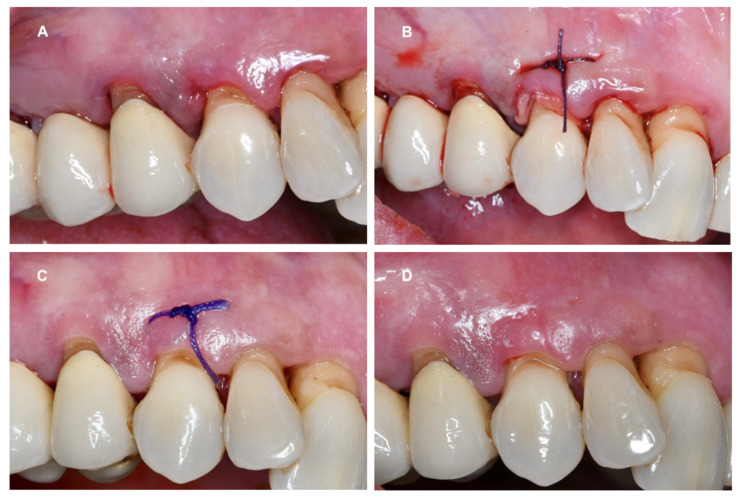
Group 2 (test) procedure: site after the SRP (**A**), PRF membrane cut and applied to the periodontal pocket and fixed with a suture (**B**), healing and suture removal after one week (**C**), and at the 6-week follow-up (**D**).

The PRF membrane was teased and carried subgingival with horizontal mattress 4-0 polyglactin 910 sutures (Vicryl; Ethicon, Somerville, NJ, USA). After one week, the suture was removed. All patients were followed up with at one week and at six weeks after the treatment (Figure 5).

**Figure 6:**
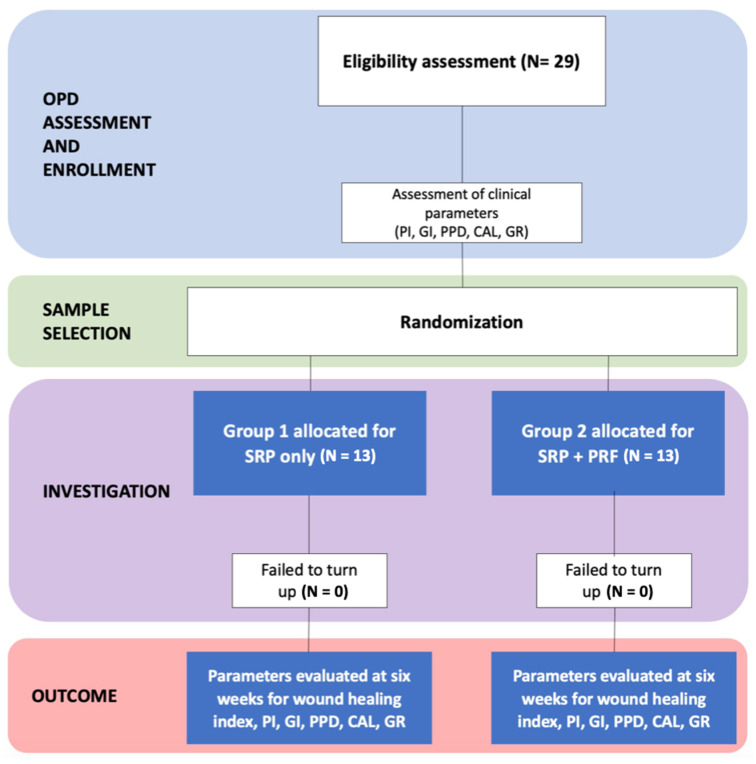
Consort flow diagram: sample size with affected sites, treatment protocol, and its outcome.

At the six-week follow-up, the CAL, PPD, GI, GR, and PI were measured and compared between the test and control groups. All participants were educated and motivated for oral hygiene maintenance with regular oral health, including interproximal brushes. Patients were recalled after one week for follow-up. In the control group, only supportive periodontal therapy was rendered. A schematic representation of the study design is provided in Figure 6 for a comprehensive overview.

### 2.6. Post-Operative Care

Patients were instructed to maintain the area plaque-free by cleaning softly using Stillman’s modified method, a soft surgical brush, and a soft interdental brush from the TePe^®^ Company (Tepe Munhygienprodukter AB, Malmö, Sweden). After a week, the suture was removed, and patients were advised to use Charter’s modified method for another five weeks. They were recalled weekly for up to six weeks, during which oral hygiene instructions were reinforced based on individual needs. Periodontal clinical parameters were then recorded at the end of the six-week period.

## 3. Results

In total, 13 patients (6 males, 7 females) with 26 periodontal pockets of 5 and 6 mm in PPD were included in this split-mouth randomized clinical trial. The average age of the patients was 29.5 (range: 30–60 years).

Table 1 shows the clinical indices at the baseline. With an independent *t* test, the pre-scaling scores between the two groups were calculated. There was no significant difference between the pre-operative parameters of the control and test groups.

Table 2 shows a comparison of the clinical indices of the 13 pockets treated with SRP (control group) with the same parameters as at the baseline. Table 3 shows a comparison between the baseline and post-operative clinical indices of the test group. In both the comparisons, there was a statistically significant improvement between the pre- and post-operative parameters of the control and test groups calculated using a paired *t* test (intragroup), suggesting a significant reduction in the periodontitis parameters with both treatment modalities (Table 2 and Table 3).

Although there were statistically significant differences with all the post-operative parameters calculated using an independent *t* test and Mann–Whitney test, these excelled far more in the test group compared to the control group (Table 4 and Table 5).

In the end, a total of 12 out of 13 pockets (92.3%) belonging to the test group closed (PPD ≤ 3 mm), while in the control group, 10 out of 13 pockets (76.9%) resolved.

## 4. Discussion

Non-surgical therapy is a blind procedure that provides the possibility of CAL gain thanks to the SRP procedure and is considered essential for the initial treatment of periodontitis [31]. Therefore, a great percentage of patients have residual pockets that should be treated using surgical procedures or tailored procedures according to the patients’ needs [31]. Non-surgical periodontal therapy, including scaling and root planing (SRP), is a fundamental initial treatment for periodontitis, often leading to clinical attachment level (CAL) gain [31]. However, residual pockets after non-surgical therapy may require surgical intervention or tailored recall protocols to address lingering issues [31]. Therefore, different approaches have been proposed to improve the non-surgical periodontal treatment [17,24,32] and to achieve the closure of pockets deeper than 5 mm. One of the most interesting novel techniques is the application of PRF during SRP therapy [17], which likely contributes more than SRP alone. Specifically, the results of the present study highlight that there was a notable reduction in PPD (2.62 ± 0.65) and a greater CAL gain (2.69 ± 0.63) in the SRP + PRF group compared to the control group at the six-week follow-up (Table 4). This statistically significant difference (*p*-value 0.001) may be associated with the rapid action of PRF in acting as a scaffold for periodontal regeneration, stabilizing the clot and preventing the migration of epithelial cells during the initial stages of healing [33,34].

However, non-significant differences (*p*-value 0.21) were observed in post-therapy gingival recession. This finding is noteworthy, as despite the use of sutures, which were reduced in size to minimize tissue tension or stress, they effectively maintained the stability of the PRF membrane within the pocket.

The stability provided by the sutures allowed the PRF membrane to remain securely in place within the pocket, promoting proper home hygiene practices. Remarkably, both study groups exhibited favorable GI and PI parameters, with even better outcomes observed in the test group compared to the control. This improvement could be attributed to the enhanced stability of the membrane ensured by the sutures, enabling patients to focus more effectively on the treated area. This represents a notable enhancement over the protocol proposed by Ozcan et al. [17], where sutures were not utilized. In that protocol, the slippery nature of PRF made it susceptible to displacement by brushing, necessitating patient instruction to refrain from brushing on the day following PRF insertion. Indeed, regardless of whether sutures were used to secure the PRF, patient compliance played a pivotal role as it was strictly mandatory for obtaining meaningful results. The effectiveness of the intervention is intricately linked to patients’ adherence to prescribed oral hygiene practices.

The test group also achieved excellent scores regarding the wound healing scale: five sites scored grade 3 (good), and eight scored grade 4 (very good), with a significant statistical difference compared to those of the control group, which scored a maximum of grade 3, but only in two sites. This difference is closely related to the amount of pocket closure, where in the test group with better healing, there was a closure rate of 92.3% compared to the control group where healing was worse.

In comparing this study’s results with the literature’s findings, it is important to note that previous studies primarily focused on the use of PRF in surgical protocols. For instance, Pradeep et al. [15] conducted a study where they combined PRF with hydroxyapatite graft for treating three-wall intrabony defects. They reported an average probing depth (PD) reduction of 3.90 ± 1.09 mm and a mean clinical attachment level (CAL) gain of 3.03 ± 1.16 mm. Similarly, Lohi et al. [35] utilized PRF with Bioactive Ceramic Composite Granules in open-flap debridement for the treatment of class II furcation defects. Their study showed a PD reduction of 3.375 ± 1.061 mm and a CAL gain of 3.00 ± 0.926 mm. Furthermore, Patel et al. [36] employed PRF in the surgical treatment of intrabony defects, achieving a significant PD reduction (3.0 ± 1.70 mm) and CAL gain (3.2 ± 1.14 mm) along with radiographic bone fill within just 6 months. These findings in the literature underscore the efficacy of PRF in promoting PD reduction, CAL gain, and bone regeneration in various periodontal procedures.

Comparing the present study’s results with those of these studies provides valuable insights into the potential of PRF in both surgical and non-surgical periodontal therapies.

Therefore, patients with 5–6 mm pockets could be efficaciously treated with a non-surgical approach in just one session of therapy, without undergoing other therapies or more invasive treatments, such as periodontal regenerative surgery.

The goal of regenerative periodontal therapy is to rejuvenate both the structure and function of the periodontium. Achieving periodontal regeneration necessitates a series of biological processes such as cell migration, adherence, growth, and differentiation, which are all crucial for enhancing the success and predictability of regenerative procedures [37]. As outlined in a position paper by the American Academy of Periodontology, regenerative techniques encompass a variety of procedures including soft tissue grafts, bone replacement grafts, root biomodifications, guided tissue regeneration, and their combinations, targeting various defects such as osseous, furcation, and recession defects [38]. Nevertheless, regenerative surgeries come with a high cost and are not without potential post-operative complications, including pain and swelling. Consequently, patients often prefer non-invasive or minimally invasive approaches [24,39].

The use of PRF in non-surgical periodontal therapy represents a promising advancement. Platelets within PRF contribute to wound healing and tissue regeneration through the release of growth factors, fostering a favorable environment for tissue repair and angiogenesis [40,41].

PRF was first developed in France by Choukroun et al. [42] for specific use in oral and maxillofacial surgery. This technique requires neither anticoagulant nor bovine thrombin (or any other gelling agent). Given the manifold benefits of PRF, including its efficacy in promoting wound healing and tissue regeneration, its antibacterial and anti-hemorrhagic properties, as well as its low risks and cost-effective preparation methods, clinicians are strongly encouraged to integrate this technology into their practice. By doing so, they can significantly enhance patient outcomes and treatment efficacy. Further, in dentistry, platelet-rich fibrin is a promising biomaterial in periodontal regeneration [43]. Anilkumar et al. [44] explored the use of PRF as an innovative approach for addressing gingival recession in the mandibular anterior region. Their study employed a combination of PRF membrane and the laterally positioned flap technique for root coverage. In contrast, Aroca et al. [45], in a randomized clinical trial, investigated the efficacy of adding PRF membrane under the modified coronally advanced flap (MCAF). They found that while this approach resulted in additional gain in gingival/mucosal thickness, it led to inferior root coverage compared to conventional therapy during a six-month follow-up period.

Additionally, PRF has been proposed to suppress cytokine release and mitigate inflammation, thus interacting with macrophages to enhance tissue healing, regeneration, and promote capillary growth [46]. The heightened concentrations of growth factors may also contribute to improved and expedited soft tissue wound healing, with observed rates that are at least two to three times faster than normal healing processes [47]. Wound healing is a complex process involving multiple cell types and growth factors. The process begins with the release of growth factors from platelets, particularly platelet-derived growth factor (PDGF), which acts as a mitogen for the proliferation of osteoblasts, endothelial cells, and mesenchymal stem cells. This ensures the restoration of microcirculation, closure of soft tissue, and expression of pro- and anti-inflammatory cytokines in gingival tissues.

However, it is noteworthy that the main advantageous effect of PRF, involving the formation of a fibrin-dense clot, could potentially contribute to the extended release of growth factors over time [21], hinting at its efficacy in long-term outcomes, such as a more substantial improvement in CAL gain, underscoring the effectiveness of PRF in achieving the closure of deeper pockets. Despite the limitations of the present study, such as the selection of pockets no deeper than 6 mm, the short follow-up period, and a relatively small but significant sample size according to the sample size calculation, the test group showed a statistically significant improvement in wound healing, achieving higher scores on the wound healing index (*p*-value < 0.001). Moreover, ethical considerations limit the detailing on histological aspects of the type of periodontal attachment; therefore, it is not possible to determine whether long junctional epithelium forms or regeneration occurs.

The potential advancements of PRF and its applications in clinical dentistry, particularly within soft tissue and bone regeneration, offer vast opportunities. However, solidifying its role in dentistry demands enhanced coherence and scientific clarity. To truly validate its clinical efficacy, it is crucial to rigorously assess various PRF preparation protocols across diverse clinical contexts. To advance our understanding and utilization of PRF, it is imperative to conduct a wider array of independent and meticulously designed randomized clinical controlled trials. Adopting a split-mouth design and integrating larger sample sizes would fortify the evidence base and ensure more robust conclusions. Independent, coherent, and scientifically validated research is essential to unlocking the full potential of PRF technology. Such validation would broaden its therapeutic applications, leading to enhanced success rates and more predictable outcomes for patients. As PRF technology is still in its nascent stages, its future impact on dentistry holds significant promise.

## 5. Conclusions

The additional application of PRF membrane alongside conventional scaling and root planing demonstrated sustainable improvements in healing outcomes. The observed advantages of this technique suggest that incorporating PRF membrane with scaling and root planing leads to reduced tissue morbidity, minimized gingival recession, and enhanced clinical parameters such as in the CAL gain and PPD reduction, potentially making the therapy more appealing to patients. Nevertheless, to gain a more comprehensive understanding of the long-term efficacy and outcomes of this procedure, further research with a larger sample size and extended follow-up periods is warranted.

## Figures and Tables

**Table 1:** Comparison of baseline pretreatment scores of each variable between two groups.

Variable at Baseline	SRP+PRF (Test)	SRP (Control)	Difference	*p* -Value
	Mean ± SD	Mean ± SD		
**CAL**	5.54 ± 0.52	5.38 ± 0.51	0.24	0.452 (NS)
**PPD**	5.31 ± 0.63	5.15 ± 0.56	0.16	0.515 (NS)
**GI**	2.62 ± 0.65	2.77 ± 0.44	−0.15	0.486 (NS)
**PI**	2.15 ± 0.38	2.38 ± 0.65	−0.61	0.282 (NS)
**GR**	0.77 ± 0.53	1.04 ± 0.78	0.27	0.312 (NS)

Independent *t* test; NS: Non-significant difference. **CAL**—Clinical Attachment Loss, **PPD**—Probing Pocket Depth, **GI**—Gingival Index, **PI**—Plaque Index, **GR**—Gingival Recession.

**Table 2:** Comparison of change in each variable within the control group (*n* = 13).

Variable of Pockets Treated with SRP (Control)	Baseline	Post-Operative	Difference	*p* -Value
	Mean ± SD	Mean ± SD		
**CAL**	5.38 ± 0.51	4.15 ± 0.69	1.23	0.001 *
**PPD**	5.15 ± 0.56	3.85 ± 0.80	1.30	0.001 *
**GI**	2.77 ± 0.44	1.77 ± 0.44	1.00	0.001 *
**PI**	2.38 ± 0.65	1.38 ± 0.65	1.00	0.001 *
**GR**	1.04 ± 0.78	0.81 ± 0.72	−0.31	0.19

Paired *t* test; * indicates a significant difference at *p* ≤ 0.05. **CAL**—Clinical Attachment Loss, **PPD**—Probing Pocket Depth, **GI**—Gingival Index, **PI**—Plaque Index, **GR**—Gingival Recession.

**Table 3:** Comparison of change in each variable within the test group (*n* = 13).

Variable of Pockets Treated with SRP + PRF (Test)	Baseline	Post-Operative	Difference	*p* -Value
	Mean ± SD	Mean ± SD		
**CAL**	5.54 ± 0.52	2.69 ± 0.63	2.85	0.001 *
**PPD**	5.31 ± 0.63	2.62 ± 0.65	2.69	0.001 *
**GI**	2.62 ± 0.65	1.08 ± 0.49	1.54	0.001 *
**PI**	2.15 ± 0.38	0.54 ± 0.52	1.71	0.001 *
**GR**	0.77 ± 0.53	0.46 ± 0.62	−0.23	0.44

Paired *t* test; * indicates a significant difference at *p* ≤ 0.05. **CAL**—Clinical Attachment Loss, **PPD**—Probing Pocket Depth, **GI**—Gingival Index, **PI**—Plaque Index, **GR**—Gingival Recession.

**Table 4:** Comparison of post-operative scores of each variable between the two groups.

Variable at Post-Operative Follow-up	SRP+PRF (Test)	SRP (Control)	Difference	*p* -Value
	Mean ± SD	Mean ± SD		
**CAL**	2.69 ± 0.63	4.15 ± 0.69	0.24	0.001 *
**PPD**	2.62 ± 0.65	3.85 ± 0.80	0.16	0.001 *
**GI**	1.08 ± 0.49	1.77 ± 0.44	−0.15	0.001 *
**PI**	0.54 ± 0.52	1.38 ± 0.65	−0.61	0.001 *
**GR**	0.46 ± 0.62	0.81 ± 0.72	0.35	0.21

Independent *t* test; * indicates a significant difference at *p* ≤ 0.05. **CAL**—Clinical Attachment Loss, **PPD**—Probing Pocket Depth, **GI**—Gingival Index, **PI**—Plaque Index, **GR**—Gingival Recession.

**Table 5:** Comparison of the wound healing index between the two groups.

Groups	Score 1	Score 2	Score 3	Score 4	Score 5	*p* Value
Test	0 (0%)	0 (0%)	5 (38.5%)	8 (61.5%)	0 (0%)	0.001 *
Control	1 (7.7%)	10 (76.9%)	2 (15.4%)	0 (0%)	0 (0%)	

Mann–Whitney test; * indicates a significant difference at *p* ≤ 0.05.

## Data Availability

The data presented in this study are available on request from the corresponding author due to privacy.
